# Distribution of allelic and genotypic frequencies of *IL1A*, *IL4*, *NFKB1* and *PAR1* variants in Native American, African, European and Brazilian populations

**DOI:** 10.1186/s13104-016-1906-9

**Published:** 2016-02-16

**Authors:** Marcos A. T. Amador, Giovanna C. Cavalcante, Ney P. C. Santos, Leonor Gusmão, João F. Guerreiro, Ândrea Ribeiro-dos-Santos, Sidney Santos

**Affiliations:** Laboratório de Genética Humana e Médica, Instituto de Ciências Biológicas, Universidade Federal do Pará, Cidade Universitária Prof. José da Silveira Netto, Rua Augusto Corrêa, 01 – Guamá, Belém, PA CEP: 66.075-110 Brazil; Núcleo de Pesquisas em Oncologia, Universidade Federal do Pará, Belém, Brazil; Laboratório de Diagnóstico por DNA, Universidade Federal do Rio de Janeiro, Rio de Janeiro, Brazil; Instituto de Patologia e Imunologia Molecular, Universidade do Porto, Porto, Portugal

**Keywords:** Polymorphisms, Cancer, Inflammatory response, Brazil, Native Americans, *IL1A* gene, *IL4* gene, *NFKB1* gene and *PAR1* gene

## Abstract

**Background:**

The inflammatory response plays a key role at different stages of cancer development. Allelic variants of the interleukin 1A (*IL1A*), interleukin 4 (*IL4*), nuclear factor kappa B1 (*NFKB1*) and protease-activated receptor 1 (*PAR1*) genes may influence not only the inflammatory response but also susceptibility to cancer development. Among major ethnic or continental groups, these polymorphic variants present different allelic frequencies. In admixed populations, such as the Brazilian population, data on distribution of these polymorphisms are limited. Here, we collected samples of cancer-free individuals from the north, northeast, midwest, south and southeast regions of Brazil and from the three main groups that gave rise to the Brazilian population: Native Americans from the Brazilian Amazon, Africans and Europeans. We describe the allelic distributions of four *IL1A* (rs3783553), *IL4* (rs79071878), *NFKB1* (rs28362491) and *PAR1* (rs11267092) gene polymorphisms, which the literature describes as polymorphisms with a risk of cancer or worse prognosis for cancer.

**Results:**

The genotypic distribution of the four polymorphisms was statistically distinct between Native Americans, Africans and Europeans. For the allelic frequency of these polymorphisms, the Native American population was the most distinct among the three parental populations, and it included the greatest number of alleles with a risk of cancer or worse prognosis for cancer. The *PAR1* gene polymorphism allelic distribution was similar among all Brazilian regions. For the other three markers, the northern region population was statistically distinct from other Brazilian region populations.

**Conclusion:**

The *IL1A*, *IL4*, *NFKB1* and *PAR1* gene polymorphism allelic distributions are homogeneous among the regional Brazilian populations, except for the northern region, which significantly differs from the other four Brazilian regions. Among the parental populations, the Native American population exhibited a higher incidence of alleles with risk of cancer or worse prognosis for cancer, which can indicate greater susceptibility to this disease. These genetic data may be useful for future studies on the association between these polymorphisms and cancer in the investigated populations.

## Background

External factors that modify tissue homeostasis, such as microorganism infection, tissue injury and exposure to contaminants may induce inflammatory processes [1]. Chronic inflammation is associated with the appearance of malignant cells and these cells’ proliferation, invasion and metastasis processes [2].

The inflammatory response may be modulated through gene expression variations due to structural genetic polymorphisms or regulatory regions. Therefore, an individual genetic composition may influence not only the inflammatory response but also susceptibility to cancer development [3, 4].

Recently, many studies have tested the association between different forms of cancer and functional variants of genes responsible for the inflammatory process [5–8], such as interleukin 1A (*IL1A*), interleukin 4 (*IL4*), nuclear factor kappa B1 (*NFKB1*) and protease-activated receptor 1 (*PAR1*) [9–12].

The gene for the pro-inflammatory cytokine *IL1A* presents an insertion/deletion (INDEL) polymorphism (3′UTR indel TTCA, rs3783553) in a binding site for the microRNAs miR-122 and miR-378 that modifies the connection between these microRNAs. The insertion (Ins) allele is related to higher gene expression [13]. Different investigations have demonstrated that individuals homozygous for the insertion (Ins/Ins) are less susceptible to developing gastric cancer, nasopharyngeal cancer, hepatocellular carcinoma and cervical cancer [13–16].

Interleukin-4 (*IL4*), which is an anti-inflammatory cytokine gene, presents a VNTR polymorphism (intron 3 VNTR—70 bp, rs79071878) located on the third intron. Different alleles for this polymorphism modulate gene expression. The allele with two repeats (A2) is related to higher gene expression, while the allele with three repeats (A3) is related to lower expression [17, 18]. A homozygous genotype for higher *IL4* expression (A2/A2) is associated with a worse prognosis in bladder cancer patients and with oral and pharyngeal cancers [19, 20].

Transcription factor NFKB1 plays an important role in the inflammatory process, and it can influence cancer development and aggressiveness, increasing tumour angiogenesis and repressing the immune response [21]. The *NFKB1* gene carries an INDEL polymorphism in the promoter region (−94 indel ATTG, rs28362491) that exerts a regulatory effect on gene expression. The insertion allele is associated with an increase in promoter activity and protein synthesis [22].

Several studies on the association between rs28362491 and the risk of developing cancer show conflicting results [9, 23–25]. In a recent meta-analysis of 21 case–control studies, Yang et al. [21] showed that the insertion allele for this polymorphism is significantly associated with a risk of developing oral, prostate and ovary cancers. The analyses also reveal similar results in Asian populations, but not in European populations, which suggests ethnic variations in predisposition to different types of cancer.

The receptor PAR1 is a member of the superfamily of G protein-coupled membrane receptors [26]. These receptors regulate processes ranging from vascular integrity to systemic inflammation. Activation of the PAR1 receptor in epithelial cells, macrophages and endothelial cells promotes the release of pro-inflammatory mediators, such as TNF, IL1β, IL2, IL6, CXCL8 and CCL2 [27]. The *PAR1* gene features an INDEL polymorphism (−506 indel—13 bp, rs11267092) located in the promoter region [28]. The deletion allele (Del) is related to a better prognosis in breast, stomach and oesophagus cancers [29–31].

The polymorphisms described above exhibit common features. (1) They are functional polymorphisms that alter the expression of genes that participate in and for metabolic pathways associated with carcinogenesis. (2) Such genes are associated with different types of cancer with high incidence in the Brazilian population, especially prostate, stomach, oesophagus, breast and cervical cancers [32]. (3) The genes vary in populations with different ethnic and geographic origins [33]. (4) Information on allele distributions in Native Americans and admixed populations, such as the Brazilian population, is limited. (5) From a technical perspective, all of the investigated polymorphisms are small INDELs; therefore, genotyping may be performed using a single PCR step followed by capillary electrophoresis, which is an accessible and low-cost laboratory technique.

The Brazilian population is one of the most heterogeneous populations worldwide that is formed by an admixture of Native Americans, Europeans and Africans. This heterogeneity has been well-documented in several genetic investigations [34–39]. The admixture process occurred through different means between the Brazilian geographic regions. Therefore, the Native American contribution is more pronounced in Northern Brazil; the African contribution is more elevated in Northeast Brazil; and the South features a European predominance with little Native American and African influence [39].

We must know the distribution of allelic frequencies of these polymorphisms [*IL*-*1A* (rs3783553), *IL4* (rs79071878), *NFKB1* (rs28362491) and *PAR1* (rs11267092)] for association studies on cancer, data for which is limited on Brazilian populations. Thus, the aim of this study is to characterize allelic distributions in a representative sample of the Brazilian population from all geographic regions of the country.

## Results

The allele and genotype frequencies of the investigated polymorphisms (rs3783553, rs79071878, rs28362491 and rs11267092) in samples from the five Brazilian geographic regions and in the parental populations are presented in Table 1. The genotypic distributions of the markers exhibited a Hardy–Weinberg equilibrium in the investigated populations, except for the *NFKB1* gene marker in the European sample (p = 0.003).

**Table 1:** Distribution of the polymorphisms in *IL*-*1A, IL4, NFKB1* and *PAR1* genes in Brazilian regions and parental populations

Genotypes	North No. (%)	Northeast No. (%)	Midwest No. (%)	Southeast No. (%)	South No. (%)	Native Americans No. (%)	Africans No. (%)	Europeans No. (%)
IL1A (rs3783553)	180	187	186	184	191	222	211	268
Ins/Ins	52 (28.9)	89 (47.6)	57 (30.6)	91 (49.5)	102 (53.4)	08 (13.5)	129 (61.1)	132 (33.7)
Ins/Del	92 (51.1)	77 (41.2)	100 (53.8)	74 (40.2)	70 (36.6)	65 (45.5)	71 (33.7)	111 (55.9)
Del/Del	36 (20.0)	21 (11.2)	29 (15.6)	19 (10.3)	19 (10.0)	149 (41.0)	11 (5.2)	25 (10.4)
Ins	0.545	0.682	0.575	0.696	0.717	0.182	0.780	0.700
Del	0.455	0.318	0.425	0.304	0.283	0.818	0.220	0.300
IL4 (rs79071878)	180	187	186	184	191	222	211	268
A3/A3	61 (33.9)	109 (58.3)	104 (55.9)	98 (53.3)	114 (60.0)	12 (5.4)	22 (10.4)	143 (53.3)
A3/A2	89 (49.4)	72 (38.5)	70 (37.6)	78 (42.4)	69 (36.3)	77 (34.7)	133 (63.0)	109 (40.7)
A2/A2	30 (16.7)	06 (3.2)	12 (6.5)	08 (4.3)	07 (3.7)	133 (59.9)	56 (26.6)	16 (6.0)
A3	0.586	0.775	0.747	0.745	0.782	0.227	0.420	0.737
A2	0.414	0.225	0.253	0.255	0.218	0.773	0.580	0.263
NFKB1 (rs28362491)	180	187	185	184	191	222	211	268
Ins/Ins	34 (18.9)	75 (40.0)	62 (33.5)	61 (33.2)	73 (38.2)	30 (13.5)	47 (22.3)	90 (33.6)
Ins/Del	99 (55.0)	80 (43.0)	88 (47.6)	88 (47.8)	93 (48.7)	101 (45.5)	109 (51.7)	151 (56.3)
Del/Del	47 (26.1)	32 (17.0)	35 (18.9)	35 (19.0)	25 (13.1)	91 (41.0)	55 (26.0)	27 (10.1)
Ins	0.464	0.615	0.573	0.571	0.626	0.363	0.481	0.617
Del	0.536	0.385	0.427	0.429	0.374	0.637	0.519	0.383
PAR1 (rs11267092)	180	187	186	183	191	222	199	267
Ins/Ins	11 (6.1)	17 (9.1)	18 (9.8)	22 (12.0)	16 (8.4)	02 (0.9)	56 (28.1)	12 (4.5)
Ins/Del	56 (31.1)	76 (40.6)	67 (36.0)	64 (35.0)	61 (31.9)	17 (7.7)	86 (43.2)	98 (36.7)
Del/Del	113 (62.8)	94 (50.3)	101 (54.3)	97 (53.0)	114 (59.7)	203 (91.4)	57 (28.7)	157 (58.8)
Ins	0.217	0.294	0.277	0.295	0.243	0.047	0.497	0.228
Del	0.783	0.706	0.723	0.705	0.757	0.953	0.503	0.772

When we compared allelic distributions in parental populations, all markers exhibited a significantly different allele frequency between the three groups, and the Native American populations exhibited more differences from the continental populations (Table 2). Among the four markers, the average frequency difference (value δ) between the Native Americans and Africans was 34 %; between the Native Americans and Europeans was 37 %; and between the Europeans and Africans was 20 %. When we considered each marker individually, a higher average frequency difference was observed between the continents for *IL1A* (40 %) followed by *IL4* (34 %), *PAR1* (30 %) and *NFKB1* (17 %).

**Table 2:** Comparison of the genotypic distribution of polymorphisms in *IL1A, IL4, NFKB1* and *PAR1* genes between the studied populations

Populations	*IL1A* (rs3783553)		*IL4* (rs79071878)		*NFKB1* (rs28362491)		*PAR1* (rs11267092)	
	χ^2^ (df = 2)	p value*	χ^2^ (df = 2)	p value*	χ^2^ (df = 2)	p value*	χ^2^ (df = 2)	p value*
Native Americans × Africans	216.025	<0.0001	54.614	<0.0001	12.663	0.0018	177.78	<0.0001
Native Americans × Europeans	207.731	<0.0001	205.587	<0.0001	70.009	<0.0001	66.495	<0.0001
Africans × Europeans	7.595	0.0224	71.667	<0.0001	22.701	<0.0001	67.497	<0.0001
North × Northeast	14.860	0.0006	31.226	<0.0001	19.925	0.0001	5.929	0.0516
North × Midwest	1.219	0.5438	21.098	<0.0001	13.136	0.0014	3.249	0.197
North × Southeast	17.801	0.0001	22.03	<0.0001	10.858	0.0044	5.287	0.0711
North × South	24.171	<0.0001	33.100	<0.0001	20.701	<0.0001	0.819	0.6641
Northeast × Midwest	11.280	0.0036	2.143	0.3435	1.085	0.5814	0.844	0.6559
Northeast × Southeast	0.158	0.4292	1.086	0.5810	1.651	0.4380	1.837	0.3992
Northeast × South	1.276	0.5284	0.259	0.8785	1.821	0.4022	3.554	0.1692
Midwest × Southeast	13.769	0.001	1.4	0.4966	0.129	0.9376	0.56	0.7556
Midwest × South	20.051	<0.0001	1.810	0.4046	1.366	0.5051	1.119	0.5715
Southeast × South	0.608	0.738	1.844	0.3976	2.277	0.3202	2.133	0.3441

* All p values were corrected by FDR method

For the geographic region comparisons (Table 2), rs11267092 (*PAR1* gene) showed no significant difference between the Brazilian regions. The distributions of the other three polymorphisms (rs3783553, rs79071878 and rs28362491) were statistically similar between the northeast, south and southeast regions.

The analyses showed statistically significant differences in the rs3783553, rs79071878 and rs28362491 polymorphism distributions between the northern population and the populations in the other regions, except the rs3783553 polymorphism in the midwest region (p = 0.543). This polymorphism exhibited a significantly different distribution in the midwest population compared with the northeast, south and southeast regions.

All polymorphisms investigated here have been previously described as associated with a predisposition to some form of cancer [14, 15, 21, 24, 25], as well as compared with the prognosis of the disease [19, 29–31]. We proposed a different approach to analysing the population data. Under this new approach, the proportion of individuals who are allele carriers are considered at risk for cancer or worse prognosis for cancer (referred to as potentially deleterious alleles [40]), including the deletion allele for rs3783553, allele A2 for rs79071878, the insertion allele for rs28362491, and the insertion allele for rs11267092.

Based on this analysis, the proportion of potentially deleterious alleles is higher in the Native American population (50 %) than the African (44 %) and European (35 %) populations. Among Brazilians, the proportion of potentially deleterious alleles is 40 %. Likewise, the proportion of individuals who are carriers of six or more alleles associated with cancer is greater among the Native Americans (8.6 %) than the Africans (5.2 %) and Europeans (1.9 %), and the proportion of Brazilians (4.2 %) is intermediate among the ancestral population values. The proportion of carriers is not evenly distributed between the geographic regions. The proportion is smaller in the Northeast Brazil population (2.7 %), intermediate in Midwest and South Brazil populations (mean = 3.4 %) and more elevated in the North (5.0 %) and Southeast Brazil populations (6.5 %).

## Discussion

This is the most comprehensive study on the rs3783553, rs79071878, rs28362491 and rs11267092 polymorphism variations in Brazil and among its ancestral populations. This is also the first study to describe the variability of these markers in Native American populations.

We evaluated the distribution of allele and genotype frequencies for these four markers among the pairs of ancestral and Brazilian populations using the Chi-square test with the due statistical corrections (false discovery rate) to avoid spurious correlations. This test is adequate to compare two or more populations for a qualitative variable [41]. Furthermore, our sample size is representative of the populations studied here (928 Brazilians, 222 Native Americans, 211 Africans and 268 Europeans) and adequate for using INDEL-type polymorphisms, which present a low mutation rate compared with other types of polymorphisms [42].

The data from the ancestral populations investigated here reveal considerable heterogeneity between continental populations. All investigated markers present δ values greater than 30 %, except for the *NFKB1* gene polymorphism. In this set of markers, the Native American populations were the most differentiated among the investigated continental populations.

Given the differences in the observed allelic distribution between the parental populations, we tested the hypothesis that these differences were due to population sampling. Therefore, we used data published in the 1000 Genomes project (Table 3). We verified that the cited frequency for the *IL1A, NFKB1* and *PAR1* markers (the three markers for which data were available) in the African and European populations are similar to the frequency observed herein. Thus, we discard the hypothesis and assume that our samples are representative of the European, African and Native American populations.

**Table 3:** Frequencies of the deletion allele in the polymorphisms of the *IL1A*, *IL4*, *NFKB1* and *PAR1* genes in Brazil and in other world populations

Populations	Number of Chromosomes	References	*IL1A*	*IL4* (A2)	*NFKB1*	*PAR1*
Brazil	1848	This study	0.358	0.273	0.426	0.737
Native Americans	444	This study	0.818	0.773	0.637	0.953
Africans	422	This study	0.220	0.580	0.519	0.503
Europeans	536	This study	0.300	0.263	0.383	0.771
Africans	2184	1000 Genomes [33]	0.230	–	0.530	–
African Americans	2184	1000 Genomes [33]	–	–	–	0.500
Europeans	2184	1000 Genomes [33]	0.330	–	0.410	0.750

Despite the frequency differences between the parental populations and the intense and heterogeneous process of admixture that formed the current Brazilian population, the allelic distribution is relatively homogeneous throughout most of the country. In general, only the northern population exhibits an allele distribution that significantly differs from the other geographic regions for three of the four investigated markers.

We understand that these differences may be explained by a greater contribution of Native Americans to the northern populations. Previous estimates using different types of genetic markers show that the greatest Native American genetic contribution among Brazilians occurs in North populations [36, 38, 39, 43]. Moreover, data generated in the present work show that Native Americans form the most differentiated group of all the continental populations that form the Brazilian population. Therefore, we believe that, conjunctively, these two factors may explain the observed differentiation in the Northern Brazil population.

Our analyses involving potentially deleterious alleles demonstrate that the Native American population presents a higher proportion (50 %) of these alleles compared with the other parental populations (44 and 35 % for African and European, respectively). This analysis holds when considering the proportion of carriers with six or more (among eight possible) potentially deleterious alleles.

However, we cannot evaluate how this genetic composition may be associated with a higher incidence of cancer in this population because Native American populations form traditional communities that reside in outlying areas of urban centres. Epidemiological data are not available on the incidence of different types of cancer among these populations.

Despite the absence of epidemiological data, recent studies demonstrate that different forms of cancer are associated with a higher (or lower) contribution from Native American ancestry in admixed populations from South America. The investigations were associated with acute lymphoblastic leukaemia [44], breast cancer [45] and gastric cancer [45–47].

## Conclusion

In summary, our study shows that the allelic distribution of the *IL*-*1A* (rs3783553), *IL4* (rs79071878), *NFKB1* (rs28362491) and *PAR1* (rs11267092) gene polymorphisms differs between European, African and Native American populations. Further, the same heterogeneity is not observed between regional populations in Brazil, except for the northern region, which significantly differs from the other four Brazilian regions.

Moreover, the results show that the Native American population includes a greater proportion of carriers with six or more alleles associated with cancer, which suggests that this population may have a higher risk of developing (or worse prognosis for) diseases associated with these alleles. The presented genetic data may be useful for future studies on the association between these polymorphisms and cancer in these populations.

## Methods

### Study population

The study population consists of 928 non-related and cancer-free adult individuals, recruited in ten Brazilian Federal Units, between the years of 2009 and 2010. In the present work, 180 individuals (90 male and 90 female) residing in the states of Pará (60), Amazonas (60) and Rondônia (60) represented the North region; 187 individuals (107 male and 80 female) residing in the states of Ceará (135) and Pernambuco (52) represented the Northeast region; 186 individuals (95 male and 91 female) residing in the states of Goiás (101), Mato Grosso do Sul (49) and Distrito Federal (36) represented the Midwest region; 184 individuals (90 male and 94 female) residing in the state of São Paulo represented the Southeast region; and 191 individuals (96 male and 95 female) residing in the state of Rio Grande do Sul represented the South region. Additional details may be found in a previous study [48].

In addition, we investigated a sample of 701 individuals representative of the main ethnic groups that originated the Brazilian population: 222 Native Americans from nine tribes of the Brazilian Amazon (Tiriyó, Waiãpi, Zoé, Urubu-Kaapor, Awa-Guajá, Parakanã, Wai Wai, Gavião, Zoró) [49]; 211 Africans (Angola, Mozambique, Congo Republic, Cameroon, Ivory Coast) [50]; and 268 Europeans (all from Portugal and Spain) that have been commonly studied in previous investigations on Brazilian ancestry [37, 39].

Informed consent for DNA analysis was obtained from healthy individuals for research purposes. Ethics approval was obtained from the local committee of Instituto de Ciências da Saúde, Universidade Federal do Pará.

### Genotyping of investigated polymorphisms

Four polymorphisms were genotyped by a single multiplex reaction with Master Mix QIAGEN^®^ Multiplex PCR kit (Qiagen, Hilden, Germany) and the primers described in Table 4.

**Table 4:** Characteristics of the investigated markers

Gene	rs	Primers sequences (5′–3′)	Polymorphism	Amplicon (bp)	Fluorochrome
*IL1A*	3783553	F-TGGTCCAAGTTGTGCTTATCC R-ACAGTGGTCTCATGGTTGTCA	INDEL—4pb	230–234	6-FAM
*IL4*	79071878	F-AGGGTCAGTCTGGCTACTGTGT R-CAAATCTGTTCACCTCAACTGC	VNTR—70 pb	217–287	HEX
*NFKB1*	28362491	F-TATGGACCGCATGACTCTATCA R-GGCTCTGGCTTCCTAGCAG	INDEL—4pb	156–160	6-FAM
*PAR1*	11267092	F-AAAACTGAACTTTGCCGGTGT R-GGGCCTAGAAGTCCAAATGAG	INDEL—13pb	265–277	HEX

Multiplex PCR products were separated and analyzed by capillary electrophoresis on the ABI 3130 Genetic Analyzer instrument (Applied Biosystems), using GS-500 LIX as pattern of molecular weight (Applied Biosystems), G5 virtual filter matrix and POP7 (Applied Biosystems). After data collect, samples were analyzed in GeneMapper^®^3.7 software (Applied Biosystems).

### Statistical analyses

Allelic and genotypic frequencies were obtained by direct counting and δ value (delta value) was determined by substracting values of allele frequency in the studied parental populations, as described by Santos et al. [39]. Hardy–Weinberg equilibrium deviations were tested in Arlequin 3.1 software [51]. Differences in genotypic frequencies between Brazilian regions and parental populations were measured by Chi-square test (χ^2^ test, df = 2) in BioEstat software [52]. FDR (False Discovery Rate) method was used to correct multiple analyses [53]. These analyses were performed in the statistical package R Calculation. *P* value was considered significant if lower than 0.05.
